# LESSONS FROM CAPABLE: DRIVERS, IMPLEMENTATION EXPERIENCE, AND MARKET READINESS CONSIDERATIONS

**DOI:** 10.1093/geroni/igac059.1294

**Published:** 2022-12-20

**Authors:** Deborah Paone, Sarah Szanton

## Abstract

External, organizational, and individual factors which drive or restrain adoption, implementation, and scaling of CAPABLE provide lessons for other programs to move from research to practice and promote sustainability. Lessons are offered for policymakers, payers, consumers, and communities to support evidence-based programs that improve function and reduce disability for older adults. This symposium presents results from a 3-year study of implementation of CAPABLE by 40 organizations, an analysis of the drivers and restrainers of CAPABLE dissemination, and a 1-year pilot study on engaging unpaid care partners in CAPABLE. We will explore the lessons from each--finding synergy and unique insights. CAPABLE is a case example to guide efforts at the organizational, system, and societal levels.

